# Traumatic Penile Pain: A Case of Dorsal Vein Thrombophlebitis after Intercourse

**DOI:** 10.1155/2018/4205628

**Published:** 2018-04-12

**Authors:** Garry J. Kennebrew, Benjamin Daggett, Reis B. Ritz

**Affiliations:** Department of Emergency Medicine, Carl R. Darnall Army Medical Center, 36065 Santa Fe Avenue, Fort Hood, TX 76544, USA

## Abstract

Dorsal Vein thrombosis, also known as Mondor's disease of the penis, is a superficial thrombophlebitis first described in the literature by Falco in 1955. Mondor's disease refers to a superficial thrombophlebitis of any locale. Diagnosis can be made clinically with palpation of a mobile, cord-like thickening on dorsum of penis without associated evidence of inflammation, infection, or dermatologic changes. Bedside ultrasonography with color Doppler can aid in the diagnosis of penile thrombophlebitis by revealing a noncompressible superficial vessel with normal surrounding flow. The following case presentation details the etiology, diagnosis, and management of a particularly rare disease process.

## 1. Introduction

Painful penile trauma can result in several clinical conditions including superficial thrombophlebitis. Mondor's disease was initially identified in the thoracoabdominal wall; however literature describes several other anatomic locations including axilla, breast, and antecubital fossa. This case details what appears to be a very rare result of penile trauma.

## 2. Case Presentation

**Figure 1:**
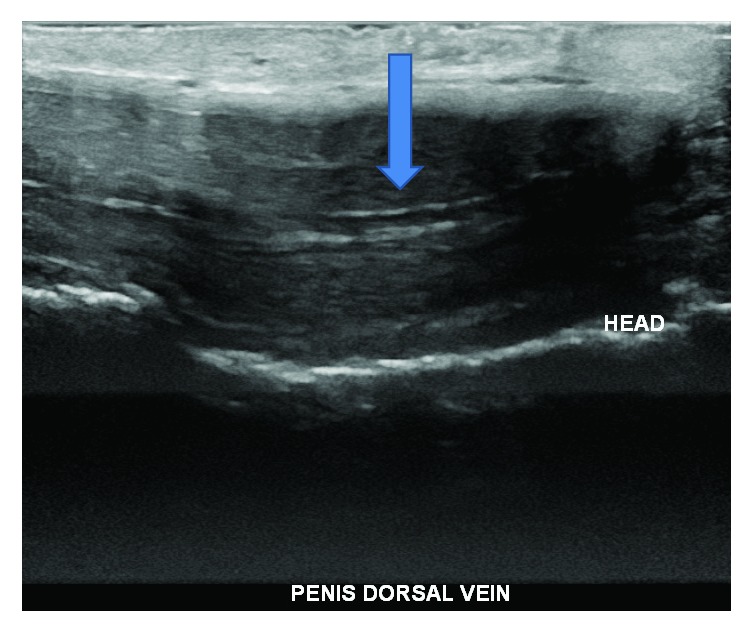
Penile ultrasound with the arrow directed towards thrombus in the dorsal vein. It is noncompressible.

**Figure 2:**
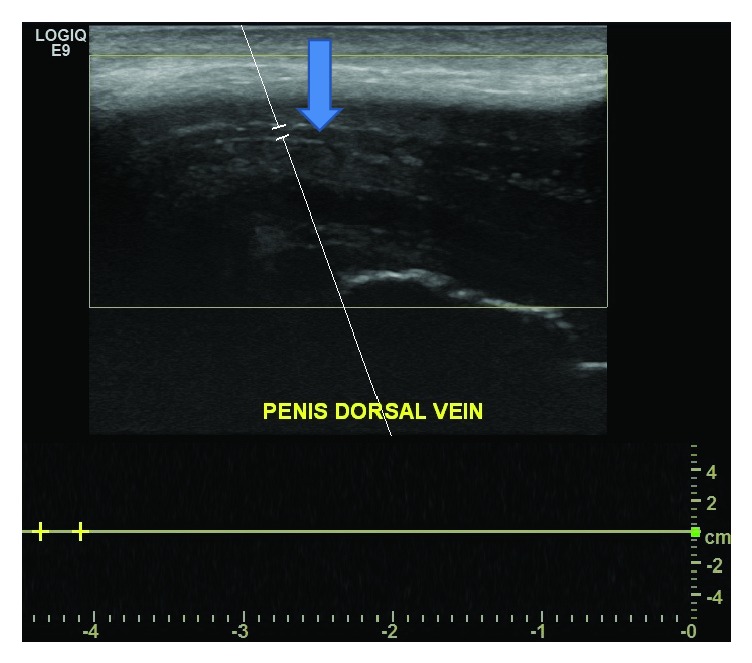
Penile ultrasound with the arrow directed towards Doppler noting absence of flow in dorsal vein.

A 31-year-old male presented to the Emergency Department (ED) for two days of penile pain, swelling, and redness beginning after intercourse. He noted swelling on the dorsum of his penis without priapism or dysuria. Review of systems was negative for abdominal pain, scrotal pain, or systemic symptoms. His initial vitals were a blood pressure of 143/87, heart rate 83, respiratory rate 20, and an oral temperature of 97.5 F. His physical exam was notable for a palpable cord and tenderness to dorsum of the penis and pubis symphysis. A complete blood count (CBC), urinalysis, and Doppler ultrasound of the penis (Figures 1 and 2) were obtained. A penile ultrasound revealed complete thrombosis of the dorsal vein. The CBC was within normal limits and urinalysis results were negative for red or white blood cells. The patient was treated with oral pain control, aspirin, and discharged home with Urology follow-up.

## 3. Discussion

Mondor's disease is named after Henri Mondor who first reported superficial thrombophlebitis in 1939 [1]. Incidence is very rare as there is a very limited number of published literature regarding Penile Mondor's disease. It is most likely underreported due to its variably benign presentation and subsequent resolution. Even with plausible underreporting, incidence remains rare. One report discusses incidence in patients presenting to an STD clinic. 18 out of 1296 patients, or approximately 1.4%, presented with various complaints and demonstrated an exam consistent with Mondor's disease. Spontaneous resolution typically occurred in 1–4 weeks.

Etiology of this disease is unknown and it is not apparent that Virchow's triad of hypercoagulability, hemostasis, and endothelial dysfunction contributes to development of penile thrombophlebitis [2]. Known causes include trauma, prolonged and or vigorous intercourse, infection, surgery, and neoplasm. Trauma and intercourse are the most reported etiologies currently documented in the literature. The presentation can also vary. Ages range from 18 to 70 and symptoms can be immediate or delayed up to 48 hrs after inciting incident [3]. It can be painful although most literature reports painless cord-like band on dorsal aspect of penis, overlying skin intact, mobile without evidence of infection or inflammation [1, 4]. Differential Diagnoses that must be entertained include penile fracture, Peyronie's disease, and sclerotizing lymphangitis. However, ultrasound can be used to aid diagnosis and findings include a noncompressible superficial vessel with normal surrounding flow. Mainstay of treatment includes symptomatic or supportive care until symptoms resolve spontaneously. These measures include pelvic/phallic rest and NSAIDs [1–5]. One particular study of 30 patients by Özkan et al. details a treatment of cefuroxime 500 mg twice daily for one week, aspirin 300 mg daily, and topical heparinoid ointment twice daily for a month. In this study 93% of patients had a normal physical exam at one month [6]. Most symptoms resolve spontaneously within 1–6 weeks and very few references recommend follow-up for a hypercoagulable workup or evaluation [1].
